# Effects of seasonal and climate variations on in-hospital mortality and length of stay in patients with type A aortic dissection

**DOI:** 10.1186/s13019-021-01639-z

**Published:** 2021-09-08

**Authors:** Zeng-Rong Luo, Zhi-Qin Lin, Liang-wan Chen, Han-Fan Qiu

**Affiliations:** 1 Department of Cardiovascular Surgery and Cardiac Disease Center, Union Hospital, Fujian Medical University, Fuzhou, 350001 People’s Republic of China; 2 Key Laboratory of Cardio-Thoracic Surgery (Fujian Medical University), Fujian Province University, Fuzhou, People’s Republic of China

**Keywords:** Seasonal, Climatic, Type A acute aortic dissection, Length of stay

## Abstract

**Objective:**

To investigate the effects of seasonal and climatic changes on postoperative in-hospital mortality and length of stay (LOS) in patients with type A acute aortic dissection (AAD).

**Methods:**

Patients undergoing implantation of the modified triple-branched stent graft to replace the descending aorta in addition to aortic root reconstruction for type A AAD in our hospital from January 2016 to December 2019 were included. Relevant data were retrospectively collected and analyzed.

**Results:**

A total of 404 patients were included in our analyses. The multivariate unconditional logistic regression analysis showed that patients admitted in autumn (OR 4.027, 95% CI 1.023–17.301, P = 0.039) or with coronary heart disease (OR 8.938, 95% CI 1.991–29.560, P = 0.049) were independently associated with an increased risk of postoperative in-hospital mortality. Furthermore, patients admitted in autumn (OR 5.956, 95% CI 2.719–7.921, P = 0.041) or with hypertension (OR 3.486, 95% CI 1.192–5.106, P = 0.035) were independently associated with an increased risk of longer LOS.

**Conclusion:**

Patients admitted in autumn or with coronary heart disease are at higher risk of in-hospital mortality following surgery for type A AAD. Also, patients admitted in autumn or with hypertension have a longer hospital LOS. In the autumn of the temperature transition, we may need to strengthen the management of medical quality after surgery for type A AAD.

## Introduction

Acute aortic dissection (AAD) is one of the cardiovascular diseases associated with the highest risk of mortality. Especially, Stanford type A AAD is life-threatening that requires emergency surgical intervention [1]. Therefore, identification of risk factors affecting the outcomes is valuable for risk stratification and prognostication. Previous studies have reported that the incidence of cardiovascular disorders including coronary heart disease, stroke, supra-ventricular tachycardia and heart failure are associated with distinct seasonal variations [2–4], with peak hospital admission reported during the winter. Also, a peak incidence of type A AAD has been observed in winter but lowest in the summer, while the average temperature on the day of patient admission with AAD is higher than that without AAD [5–8]. Nevertheless, evidence is lacking on the association of seasonal and climate variations with in-hospital mortality and length of stay (LOS) in postoperative patients with AAD, which formed the basis of this study.

## Methodology

This study was approved by the ethics committee of Fujian Medical University, China.

### Patients and outcome measures

Patients undergoing implantation of the modified triple-branched stent graft to replace the descending aorta in addition to aortic root reconstruction for type A AAD in hospitals of Fujian province, China from January 2016 to December 2019 were included. Retrospective data collection was conducted, which included patients’ age, gender, co-morbidities such as diabetes, hypertension, coronary heart disease, chronic obstructive pulmonary disease (COPD), renal dysfunction, malperfusion syndromes, admission route, history of weekend surgery, onset time of AAD and LOS. A database with Excel software was created by another researcher for data input and verification.

The Fujian province in China has a typical southern hemisphere temperate monsoon climate with four distinct seasons. Meteorological data during our study period were obtained from the Fujian Meteorological Bureau, containing the records of the daily minimum temperature, the average minimum temperature, the daily temperature difference, the average daily temperature difference and the average air quality index (AQI). All the data were collected by a single researcher. A database with Excel software was created by another researcher for data input and verification. The classification of seasons was as follows: autumn (21st September–20th December), winter (21st December–21st March), spring (22nd March to 21st June 21), and summer (22nd June–20th September). The temperatures were categorized into < 11 °C, 11–16 °C, 16–23 °C and > 23 °C based on the quartile of the average minimum temperature. Outcome indicators were postoperative in-hospital mortality and LOS of survivors.

### Surgical procedure for AAD

**Fig. 1:**
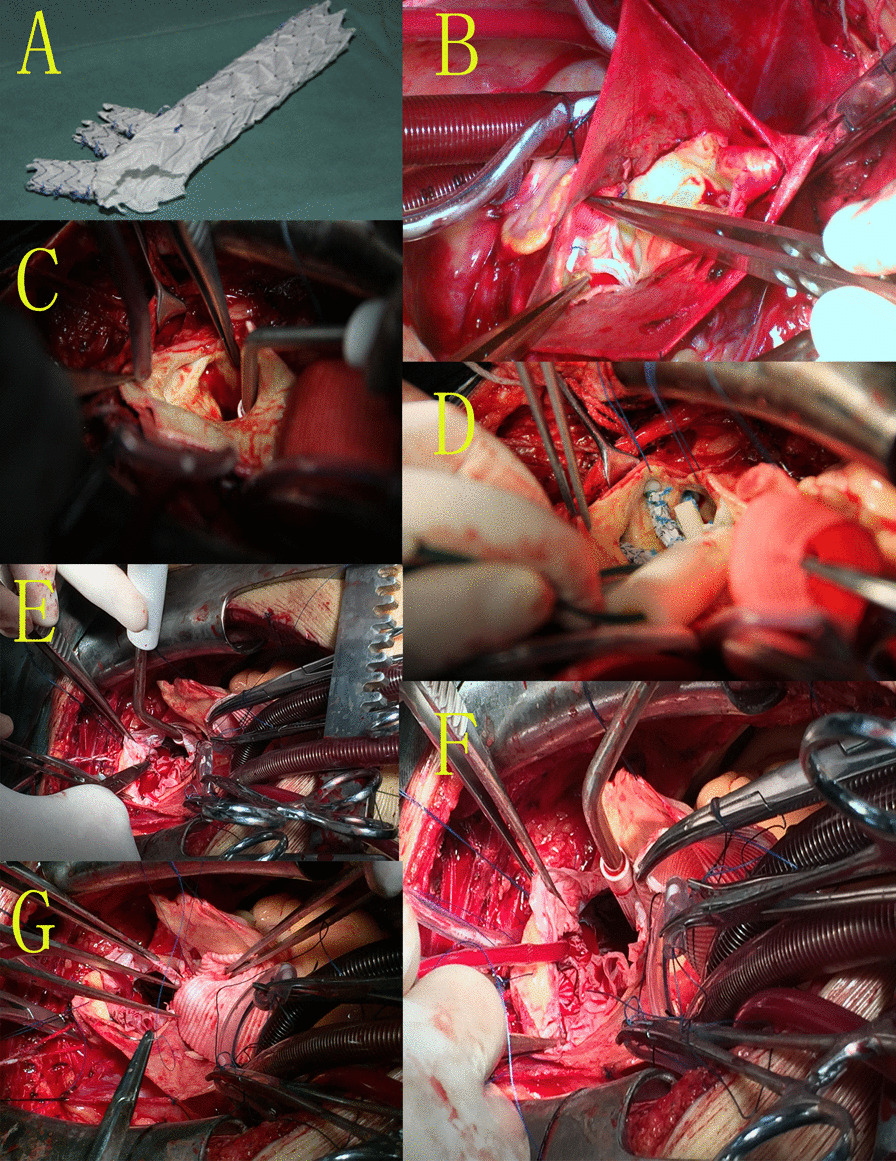
The implantation of the modified triple-branched stent graft for the replacement of the descending aorta combined with aortic root reconstruction. **a** Modified triple-branched stent-graft; **b** aortic root reconstruction and replacement of the ascending aorta with artificial graft; **c** exposure of the branching arteries in the aortic arch; **d** implantation and release of the modified triple-branched stent-graft; **e** Two-branch stent grafts were secured appropriately; **f** a perfusion tube was inserted into the left carotid artery via the second branch of the stent graft, and bilateral antegrade cerebral perfusion was performed; **g** the artificial graft was continuously sutured with the proximal end of the modified triple-branched stent graft

The surgical procedure for AAD included the implantation of a modified triple-branched stent graft for the replacement of the descending aorta combined with aortic root reconstruction as described previously [9–11] and illustrated in Fig. 1 (A-G).

### Statistical analysis

Analyses were performed using the IBM SPSS Statistics 23.0 software. Demographics of patients were described as percentages and means with standard deviations. A cross-sectional analysis was performed on variables associated with in-hospital mortality and LOS. The chi-square test was used to analyze categorical variables, while the analysis of variance (ANOVA) was used for two or more grouped variables. To explore the association of continuous variables with in-hospital mortality and LOS, the Tukey’s post hoc range test was used to compare the means of each group to arrive at a significant ANOVA. Non-normally distributed variables were analyzed with non-parametric tests (Mann–Whitney U test and Kruskal–Wallis H test). Univariate and multiple logistic regression analyses were conducted to explore the relationship of seasonal and climatic variations with in-hospital mortality and demographic covariates of patients. A further multiple regression analysis was performed by using the same covariates that were used in the above multiple logistic regression analysis to explore the predictors of LOS in postoperative survivors. Data on LOS appeared positively skewed and kurtotic. Hence, logarithmic transformation of LOS data was performed to correct the non-normality, and the power was returned to the geometric mean with an appropriately reported 95% confidence interval (95% CI). A P-value of < 0.05 was considered statistically significant.

## Result

### Patient demographics

A total of 404 patients were included in our analyses. Of these, the average age was 52.96 ± 11.73 years, 74.3% were male, 72% patients suffered from hypertension, 2.2% suffered from diabetes, 7.9% suffered from coronary heart disease, 0.7% suffered from COPD, 20.3% suffered from renal dysfunction and 17.8% suffered from malperfusion syndromes. There were 18 patients (4.5%) who died before discharge from the hospital, of which 14 (77.8%) were males.

### Risk factors associated with in-hospital mortality

The chi-square test revealed that patients admitted in autumn were significantly associated with an increased risk of in-hospital mortality (P = 0.026). Other climatic variables were not significantly associated with in-hospital mortality, including the average minimum temperature on the day of AAD onset (P = 0.066), average daily temperature difference (P = 0.605), and average AQI (P = 0.720). Patients with coronary heart disease (P = 0.000), renal dysfunction (P = 0.000) and malperfusion syndromes (P = 0.043) were also significantly associated with the risk of in-hospital mortality (Table 1).

**Table 1:** The Chi-square test of covariates for in-hospital mortality

Covariates	Survive	Death	χ^2^	P
*Age (year)*				
18–44	91 (97.8%)	2 (2.2%)	3.057	0.383
45–59	165 (93.8%)	11 (6.3%)		
60–74	119 (96.0%)	5 (4.0%)		
75–89	11 (100.0%)	0 (0.0%)		
*Gender*				
Male	300 (95.5%)	14 (4.5%)	0.000	1.000
Female	86 (95.6%)	4 (4.4%)		
*Hypertension*				
No	107 (94.7%)	6 (5.3%)	0.269	0.604
Yes	279 (95.9%)	12 (4.1%)		
*Coronary heart disease*				
No	357 (96.0%)	15 (4.0%)	20.381	< 0.000
Yes	24 (75.0%)	8 (25.0%)		
*Diabetes*				
No	378 (95.7%)	17 (4.3%)	0.958	0.328
Yes	8 (88.9%)	1 (11.1%)		
*COPD*				
No	383 (95.5%)	18 (4.5%)	0.141	0.707
Yes	3 (100.0%)	0 (0.0%)		
*Renal dysfunction*^a^				
No	300 (93.2%)	22 (6.8%)	24.230	< 0.000
Yes	61 (74.4%)	21 (25.6%)		
*Malperfusion syndromes*				
No	303 (91.3%)	29 (8.7%)	4.082	0.043
Yes	60 (83.3%)	12 (16.7%)		
*Average minimum temperature on the day of onset (*°C*)*				
< 11.00	83 (98.8%)	1 (1.2%)	7.208	0.066
[11.00, 16.00]	112 (95.7%)	5 (4.3%)		
(16.00–23.00]	98 (97.0%)	3 (3.0%)		
> 23.00	93 (91.2%)	9 (8.8%)		
*Average daily temperature difference (*°C*)*				
< 5.00	75 (97.4%)	2 (2.6%)	1.848	0.605
[5.00, 7.00]	136 (93.8%)	9 (6.2%)		
(7.00, 9.00]	105 (96.3%)	4 (3.7%)		
> 9.00	70 (95.9%)	3 (4.1%)		
*Air Quality Index, AQI*				
0–50	244 (94.9%)	13 (5.1%)	0.658	0.720
51–100	140 (96.6%)	5 (3.4%)		
101–150	2 (100.0%)	0 (0.0%)		
Spring	74 (93.7%)	5 (6.3%)	9.225	0.026
Season*				
Summer	69 (90.8%)	7 (9.2%)		
Autumn	108 (87.8%)	15 (12.2%)		
Winter	123 (97.6%)	3 (2.4%)		
*Weekend surgery history*				
No	313 (95.7%)	14 (4.3%)	0.122	0.727
Yes	73 (94.8%)	4 (5.2%)		
*Admission route*				
Outpatient admission	13 (92.9%)	1 (7.1%)	0.000	0.496
Emergency admission	372 (95.4%)	18 (4.6%)		

^*^Season: autumn (21st September–20th December), winter (21st December–21st March), spring (22nd March–21st June 21), and summer (22nd June–20th September)

^a^Defined as preoperative creatinine greater than 1.5 mg/dL

The univariate unconditional logistic regression analysis revealed that admissions in autumn but not other climatic effects were associated with a significantly increased risk of in-hospital mortality (OR 4.159, 95% CI 1.042–16.604). In addition, patients with coronary heart disease (odds ratio OR 6.899, 95% CI 3.598–38.583) and those admitted as an emergency (OR 4.488, 95% CI 1.074–5.655) were also significant risk factors for in-hospital mortality (Table 2).

**Table 2:** The univariate unconditional logistic regression analysis of covariates for in-hospital mortality

Covariates	Survive	Death	OR (95% CI)
*Age (year)*			
18–44	91 (97.8%)	2 (2.2%)	1.000
45–59	165 (93.8%)	11 (6.3%)	3.033 (0.658, 13.984)
60–74	119 (96.0%)	5 (4.0%)	1.912 (0.363, 10.079)
75–89	11 (100.0%)	0 (0.0%)	–
*Gender*			
Male	300 (95.5%)	14 (4.5%)	1.000
Female	86 (95.6%)	4 (4.4%)	1.003 (0.322, 3.127)
*Hypertension*			
No	107 (94.7%)	6 (5.3%)	1.000
Yes	279 (95.9%)	12 (4.1%)	0.767 (0.281, 2.095)
*Coronary heart disease*			
No	357 (96.0%)	15 (4.0%)	1.000
Yes	24 (75.0%)	8 (25.0%)	6.899 (3.598, 38.583)
*Diabetes*			
No	378 (95.7%)	17 (4.3%)	1
Yes	8 (88.9%)	1 (11.1%)	2.779 (0.329, 23.503)
*COPD*			
No	383 (95.5%)	18 (4.5%)	1.000
Yes	3 (100.0%)	0 (0.0%)	–
*Renal dysfunction*^a^			
No	300 (93.2%)	22 (6.8%)	1.000
Yes	61 (74.4%)	21 (25.6%)	1.066 (0.537, 4.998)
*Malperfusion syndromes*			
No	303 (91.3%)	29 (8.7%)	1.000
Yes	60 (83.3%)	12 (16.7%)	2.033 (0.155, 5.684)
*Average minimum temperature on the day of onset (*°C*)*			
< 11.00	83 (98.8%)	1 (1.2%)	1.000
[11.00, 16.00]	112 (95.7%)	5 (4.3%)	3.705 (0.425, 32.314)
(16.00–23.00]	98 (97.0%)	3 (3.0%)	2.541 (0.259, 24.890)
> 23.00	93 (91.2%)	9 (8.8%)	8.032 (0.996, 64.750)
*Average daily temperature difference (*°C*)*			
< 5.00	75 (97.4%)	2 (2.6%)	1.000
[5.00, 7.00]	136 (93.8%)	9 (6.2%)	2.482 (0.523, 11.785)
(7.00, 9.00]	105 (96.3%)	4 (3.7%)	1.429 (0.255, 8.002)
> 9.00	70 (95.9%)	3 (4.1%)	1.607 (0.261, 9.905)
*Air Quality Index, AQI*			
0–50	244 (94.9%)	13 (5.1%)	1.000
51–100	140 (96.6%)	5 (3.4%)	0.670 (0.234, 1.920)
101–150	2 (100.0%)	0 (0.0%)	–
Season*			
Spring	74 (93.7%)	5 (6.3%)	1.000
Summer	69 (90.8%)	7 (9.2%)	2.770 (0.643, 11.930)
Autumn	108 (87.8%)	15 (12.2%)	4.159 (1.042, 16.604)
Winter	123 (97.6%)	3 (2.4%)	1.025 (0.203, 5.179)
*Weekend surgery history*			
No	313 (95.7%)	14 (4.3%)	1.000
Yes	73 (94.8%)	4 (5.2%)	1.225 (0.392, 3.831)
*Admission route*			
Outpatient admission	13 (92.9%)	1 (7.1%)	1.000
Emergency admission	372 (95.4%)	18 (4.6%)	4.488 (1.074, 5.655)

^*^Season: autumn (21st September–20th December), winter (21st December–21st March), spring (22nd March–21st June 21), and summer (22nd June–20th September)

^a^Defined as preoperative creatinine greater than 1.5 mg/dL

The multivariate unconditional logistic regression analysis revealed that admission in autumn (OR 4.027, 95% CI 1.023–17.301, P = 0.039) and coronary heart disease (odds ratio OR 8.938, 95% CI 1.991–29.560, P = 0.049) were independent risk factors for in-hospital mortality (Table 3).

**Table 3:** The multivariate unconditional logistic regression analysis of covariates for in-hospital mortality

Covariates	β	Wald χ^2^	P value	OR	95%CI
Coronary heart disease	3.396	5.774	0.049	8.938	(1.991, 29.560)
Autumn	0.986	3.264	0.039	4.027	(1.023, 17.301)

### Factors associated with hospital LOS after surgery

The univariate analysis revealed that factors including hypertension (P = 0.012) and admission in autumn (P = 0.046) were significantly associated with the hospital LOS (Table 4).

**Table 4:** The Mann–Whitney U test and Kruskal–Wallis H test analysis of covariates for LOS

Covariates	Cases (%)	M	(P_25_,P_75_)	χ^2^/Z	P
*Age (year)*					
18–44	93 (23.0%)	18	(14, 24)	5.008	0.171
45–59	176 (43.6%)	18	(15, 26)		
60–74	124 (30.7%)	20.5	(16, 27)		
75–89	11 (2.7%)	21	(17, 26)		
*Gender*					
Male	90 (22.3%)	20	(15, 26)	− 0.203	0.839
Female	314 (77.7%)	19	(15, 25)		
*Hypertension*					
No	113 (28.0%)	17	(14, 23)	− 2.502	0.012
Yes	291 (72.0%)	20	(15, 27)		
*Coronary heart disease*					
No	396 (98.0%)	19	(15, 25)	− 0.794	0.427
Yes	8 (2.0%)	25	(14, 43)		
*Diabetes*					
No	395 (97.8%)	19	(15, 25)	− 0.107	0.915
Yes	9 (2.2%)	20	(16, 27)		
*COPD*					
No	401 (99.3%)	19	(15, 26)	− 1.418	0.156
Yes	3 (0.7%)	14	(8, 20)		
*Renal dysfunction*^a^					
No	322 (79.7%)	23	(18, 24)	− 0.609	0.596
Yes	82 (20.3%)	16	(13, 26)		
*Malperfusion syndromes*					
No	332 (82.2%)	18	(15, 23)	− 0.228	0.395
Yes	72 (17.8%)	18	(15, 22)		
*Average minimum temperature on the day of onset (*°C*)*					
< 11.00	84 (20.8%)	19	(15, 27)	4.502	0.212
[11.00, 16.00]	117 (29.0%)	19	(15, 24)		
(16.00–23.00]	101 (25.0%)	18	(15, 23)		
> 23.00	102 (25.2%)	22	(16, 31)		
*Average daily temperature difference (*°C*)*					
< 5.00	77 (19.1%)	20	(14, 25)	2.100	0.552
[5.00, 7.00]	145 (35.9%)	18	(15, 26)		
(7.00, 9.00]	109 (27.0%)	19	(15, 24)		
> 9.00	73 (18.1%)	20	(17, 27)		
*Air Quality Index, AQI*					
0–50	257 (63.6%)	19	(14, 25)	0.647	0.724
51–100	145 (35.9%)	19	(15, 26)		
101–150	2 (0.5%)	18	(17, 18)		
Season*					
Spring	79 (19.6%)	19	(15, 25)	7.975	0.046
Summer	76 (18.8%)	18	(14, 24)		
Autumn	123 (30.4%)	23	(16, 33)		
Winter	126 (31.2%)	19	(14, 25)		
*Weekend surgery history*					
No	327 (80.9%)	19	(15, 27)	− 0.085	0.933
Yes	77 (19.1%)	21	(14, 24)		
*Admission route*					
Outpatient admission	14 (3.5%)	17	(15, 23)	− 1.708	0.343
Emergency admission	390 (96.5%)	23	(18, 27)		

^a^Defined as preoperative creatinine greater than 1.5 mg/dL

^*^Season: autumn (21st September–20th December), winter (21st December–21st March), spring (22nd March–21st June 21), and summer (22nd June–20th September)

In the multiple regression analysis, patients admitted in autumn (OR 5.956, 95% CI 2.719–7.921, P = 0.041) or with hypertension (OR 3.486, 95% CI 1.192–5.106, P = 0.035) were independently associated with longer hospital LOS (Table 5).

**Table 5:** Multivariate linear regression analysis of covariates for LOS

Covariates	Unstandardized coefficients B	Standard error	Standardized coefficients Beta	t	P	95%CI
Hypertension	2.649	1.250	0.105	2.119	0.035	(1.192, 5.106)
Autumn	1.368	0.762	0.886	1.756	0.041	(2.719, 7.921)

## Discussion

The occurrence of AAD is multifactorial but primarily associated with three key factors: weakened aortic wall, damaged vascular endothelial resulting in the formation of the vascular endothelial slap, and the extension of endothelial damage due to hypertension [12]. Previous studies have demonstrated the relationship between numerous cardiovascular disorders with seasonal and climatic variations, including external environmental factors such as temperature and UV radiation, lifestyle factors such as diet, obesity, exercise and smoking, and other factors such as blood pressure, serum cholesterol, glucose tolerance, coagulation, acute and chronic infections [13, 14]. All these risk factors are more common in the winter and may account for seasonal variations in the incidence of AAD [15, 16]. Several studies have also suggested a possible seasonal effect on the onset of AAD, given an observed higher incidence of AAD in the winter and a lower incidence of AAD in the summer [5–8]. During the winter, the human sympathetic nervous system is activated with an increased catecholamine secretion to cope with the low temperature, resulting in increased heart rate, peripheral vascular resistance, and systemic blood pressure. High blood pressure then leads to increased blood friction against the vascular wall and surface shear stress, which enhances the occurrence and rupture of aortic dissection [17]. To date, limited studies have explored the impacts of seasonal and climatic variations on the postoperative outcomes of AAD patients.

A significant increase in the incidence of non-accidental mortality in the general population has been reported during the transitional season. A study has demonstrated a higher mortality rate during the seasonal transition from hot to cold (autumn) than that from cold to hot (spring) in Russia [18]. Also, large diurnal temperature variation leading to increased risk of cardiac mortality in autumn has also been observed [19, 20]. Unlike the regular seasonal and climatical patterns in the incidence of AAD, our univariate and multivariate regression analysis revealed patients with onset of AAD in autumn were significantly associated with a higher risk of mortality, although no significant link between other climatic factors and in-hospital mortality, such as the average minimum temperature on the day of onset of AAD, average daily temperature difference and average AQI. Numerous studies have shown that seasonal meteorological variations affect blood pressure readings in hypertensive and normotensive individuals [21–23]. In China, the weather becomes cool and humid in autumn due to frequent rainfalls and the atmospheric pressure (AP) fluctuates intensely [24]. The reduction in temperature and fluctuation in AP may lead to increased sympathetic activity, resulting in increased vasoconstriction or vasospasm and higher blood pressure consequently [25]. Also, the fluctuation of AP may increase the risk of rupture of the abdominal aortic aneurysm [26, 27]. Similarly, drastic fluctuations of systolic blood pressure in patients with AAD as a result of an increased alteration of AP in autumn may cause a greater risk of postoperative rupture of residual endothelial dissection, leading to a significantly higher in-hospital mortality rate in our study. Alternatively, other meteorological risk factors such as humidity, rainfall, etc. may also contribute to the impacts of seasonal and climatic variations on the prognosis of patients [28]. On the other hand, our analyses also revealed that coronary heart disease and admissions from outpatient settings were adverse factors associated with an increase of postoperative in-hospital mortality, suggesting that timely intervention of coronary heart disease and emergency surgery may reduce postoperative mortality.

Our analyses have also revealed that factors including the onset of AAD in autumn and hypertension were independent risk factors associated with a prolonged hospital LOS. Understandably, due to the significantly higher in-hospital mortality rate in autumn, the corresponding severe postoperative patients resulted in the increased LOS.

### Limitations

There were limitations to our study. Firstly, our patient cohort was from a single center, which therefore lacked external validity. Secondly, given the retrospective nature of our study design, the association of seasonal and climatic variation with in-hospital mortality and LOS might also be affected by other confounding variables.

## Conclusion

In patients undergoing surgery for AAD, factors including admission in autumn, coronary heart disease, renal dysfunction, malperfusion syndromes and admission from outpatient settings are associated with increased risk of postoperative in-hospital mortality. Meanwhile, patients with hypertension and admitted in autumn have longer hospital LOS following surgery for AAD. The temperature transition in autumn may be a factor that increases the risk of in-hospital mortality from type A AAD, especially in patients complicated with coronary heart disease, renal dysfunction, malperfusion syndromes. Urgent surgery for type A AAD is very necessary. Nevertheless, further studies are warranted to validate our findings.

## Data Availability

Data sharing not applicable to this article as no data sets were generated or analyzed during the current study.

## Abbreviations

LOS: Length of stay
AAD: Acute aortic dissection
AP: Atmospheric pressure
AQI: Air quality index
COPD: Chronic obstructive pulmonary disease
